# Parenting and Child/Adolescent Development: Current Updates and Global Perspectives

**DOI:** 10.3390/children12101368

**Published:** 2025-10-10

**Authors:** Elitsa Dimitrova, Apolinaras Zaborskis

**Affiliations:** 1 Institute for Population and Human Studies, Bulgarian Academy of Sciences & Plovdiv University Paisii Hilendarski, 1000 Sofia, Bulgaria; 2 Department of Preventive Medicine & Health Research Institute, Faculty of Public Health, Medical Academy, Lithuanian University of Health Sciences, A.Mickevičiaus 9, LT-44307 Kaunas, Lithuania; apolinaras.zaborskis@lsmu.lt

This Special Issue, titled “Parenting and Child/Adolescent Development: Current Updates and Global Perspectives”, brings together a diverse collection of ten articles that examine the complex challenges and opportunities shaping children’s and adolescents’ lives today. Despite addressing varied topics and contexts, the articles converge on several cross-cutting themes: the central role of the family in development, the dynamic balance between risk and protective factors, the necessity of cultural sensitivity, the value of interventions that strengthen families, and the importance of broader systemic supports. Expanding on these themes in light of both the contributions in this issue and the wider scholarly literature offers a more nuanced understanding of how children’s lives unfold within their developmental contexts.

## 1. Introduction

Family dynamics, parenting practices, and parent/caregiver–child relationships have a formative influence on children’s health, well-being, and socioemotional, cognitive, and neurobiological development, with effects that extend into later life [1,2]. Family connectedness, communication, and relationships with parents and relatives not only provide important role models but also shape identity formation and the adoption of skills and behaviors during adolescence [3,4]. For adolescents in particular, connections with family, peers, and community play a critical role in their development, as argued by Robert Blum and colleagues [5]. Supportive networks of parents, peers, and teachers have been shown to improve adolescent mental and behavioral health, with lasting benefits across the life course [6,7]. By contrast, youth development programs that frame adolescents as problems to be fixed tend to be ineffective, yet governments and non-governmental organizations continue to invest in them [8].

The evidence reviewed above demonstrates that family dynamics and relationships are central to child and adolescent development across multiple domains—including health, well-being, socioemotional functioning, cognition, and neurobiological processes. Importantly, their influence extends across diverse cultural, social, and economic contexts and is not confined to Western, high-income settings. Alongside this recognition, scholarship increasingly emphasizes the interconnectedness of ecological systems—families, peers, schools, and communities—and the ways in which these overlapping spheres interact to collectively shape developmental trajectories.

At the same time, a substantial body of research continues to document the limited effectiveness of punitive or deficit-oriented interventions that frame young people primarily as problems to be managed [8]. These findings have fueled momentum toward evidence-based approaches that promote positive youth development, emphasizing strengths, agency, and supportive environments. This global reorientation reflects not only a rejection of narrow, problem-focused models but also a paradigm shift toward holistic, contextually grounded strategies. Such approaches acknowledge the diversity of adolescent experiences, highlight the critical role of relational and ecological supports, and position adolescents as active partners in shaping their own health, well-being, and future prospects [5,9,10].

The articles published in this Special Issue respond directly to current challenges in the field facing children, adolescents, and families today. Accordingly, this editorial article seeks to organize them around several cross-cutting themes that address both enduring concerns and emerging opportunities. Together, these contributions are expected not only to deepen our understanding of how development unfolds across diverse contexts but also to illuminate innovative pathways for advancing research, informing practice, and shaping policy.

## 2. The Family as a Central Developmental Context

Family processes are consistently shown to be pivotal in shaping developmental trajectories. Jeong and Bang (contribution 1) highlight how adolescents in immigrant marriage families often provide “unavoidable family assistance, even under pressure.” Their experiences reveal both empathy toward immigrant mothers and stress when paternal involvement is diminished. Similarly, Chan et al. (contribution 2) emphasize that parental “emotion talk” significantly shapes preschoolers’ expression of anger, sadness, and positive emotions, supporting the notion that parental practices are central to children’s socioemotional development.

The literature broadly confirms that families constitute the primary ecological system in Bronfenbrenner’s (1979) Ecological Systems Theory [11]. Parenting behaviors, family communication, and emotional climates all affect how children regulate emotions, develop social skills, and manage stress [12]. Positive co-parenting, as Seijo et al. (contribution 3) argue, is associated with “healthy development of children,” reinforcing that family harmony is a cornerstone of resilience. Conversely, conflict, neglect, or imbalanced role expectations, as seen in both immigrant families and foster care transitions, may create stressors with long-term consequences.

## 3. The Interplay of Risk and Protective Factors

A second unifying theme is the constant interplay between vulnerabilities and buffers. Nesmith (contribution 4) shows that foster youth aging out of care are at risk of “homelessness and social disconnection,” yet the Bridges Transitions Framework fostered moderate to high levels of social support and higher school enrollment compared with state averages. Similarly, Valentic et al. (contribution 5) reveal that “maternal monitoring” strongly protects Croatian adolescents against substance use and bullying, while Dimitrova and Alexandrova-Karamanova (contribution 6) demonstrate that parental unemployment and economic stress increased Bulgarian adolescents’ health risk behaviors during the pandemic.

This duality is echoed in developmental criminology. Zúñiga et al. (contribution 7) identify biological, socioemotional, and contextual risk factors that elevate the likelihood of transgressive behavior, while protective factors include “cognitive, socioemotional, and personality development aspects.” Together, these findings confirm Masten’s (2001) seminal argument that resilience is “ordinary magic,” emerging when protective processes such as supportive relationships, self-regulation, and positive identity buffer the effects of adversity [13].

Protective factors often work relationally: Hadar (contribution 8) demonstrates how music therapy empowered displaced families to rediscover a “sense of agency… through controlling the musical environment.” This aligns with Bowlby’s (1969) Attachment Theory, which emphasizes the role of secure caregiver–child bonds in mitigating trauma [14]. Risk and protection, therefore, must be viewed not as static traits but as dynamic processes embedded in everyday interactions.

## 4. Cultural and Contextual Sensitivity

Across contexts, the studies demonstrate that children’s development is inseparable from cultural expectations and structural realities. Chan et al. (contribution 2) found cultural differences between Chinese American and Mexican American preschoolers: Mexican American children expressed more anger and sadness, reflecting cultural norms around emotional expression. Jeong and Bang (contribution 1) emphasize that Korean adolescents in immigrant families felt a “natural” obligation to support their mothers, shaped by Confucian traditions of filial duty and gender role expectations.

Correia et al. (contribution 9) in Cape Verde show how a family education program enhanced parental competence, but the degree of benefit varied across clusters, suggesting that cultural beliefs and parenting profiles mediate outcomes. Likewise, Seijo et al. (contribution 3) adapted the Coparenting Relationship Scale for Spanish parents, demonstrating the necessity of ensuring psychometric tools are culturally valid.

The literature reinforces this need for cultural grounding. Harkness and Super’s (1994) developmental niche framework argues that cultural settings shape parental ethnotheories, daily routines, and physical environments, which in turn shape child development [15]. Without attending to these cultural lenses, interventions risk being ineffective or even harmful [16].

## 5. The Importance of Interventions That Strengthen Families

Another shared theme is the promise of interventions that empower families rather than targeting children in isolation. Hadar (contribution 8) illustrates how dyadic music therapy “restored the children’s freedom of play” and parents’ sense of competence. Correia et al. (contribution 9) show that the Family Education and Support Program promoted positive parenting practices and increased parental efficacy. Nesmith (contribution 4) demonstrates that integrating a structured transition framework into foster care supports internal coping and social support among youth leaving care.

These interventions reflect a paradigm shift toward systemic and relational approaches. Child–Parent Psychotherapy [17,18] has long emphasized the dyad as the unit of intervention, while Triple P [19,20] and other evidence-based parenting programs highlight the role of parental self-efficacy in promoting child well-being. The findings across the ten studies support the view that strengthening parental skills, fostering supportive co-parenting, and providing structured guidance during transitions yield protective ripple effects for children.

## 6. The Role of Broader Systems

Finally, the studies highlight how families are embedded in larger systems—schools, communities, and governments—that profoundly affect child outcomes. Groff et al. (contribution 10) found that “live online classes protected parents and children” during homeschooling, while a lack of adequate support compounded stress. Dimitrova and Alexandrova-Karamanova (contribution 6) show how macroeconomic shocks such as “income reduction and temporary lay-offs” cascaded into adolescent substance use. Zúñiga et al. (contribution 7) emphasize that risk and protective factors extend to “family, school, peers, socioeconomic situation and governance,” requiring multi-level interventions.

This resonates with Bronfenbrenner’s ecological model [11], which situates the child within interconnected systems from the microsystem of family to the macrosystem of policy and culture. Public health crises like COVID-19 and structural conditions like unemployment demonstrate that no family operates in isolation. Research on social determinants of health [21] further underscores how inequities in income, housing, and education shape child outcomes long before individual-level interventions take place.

## 7. Conclusions

In synthesizing these ten studies, five cross-cutting themes stand out. Families remain the central developmental context, but their ability to nurture is shaped by the interplay of risk and protective factors, the cultural milieu in which they operate, the strength of interventions that empower them, and the broader systems within which they are embedded. Together, the evidence paints a picture of child and adolescent development as a dynamic process—shaped by both vulnerability and resilience, grounded in culture, and profoundly relational.

To move forward, research and practice must adopt an integrated perspective: one that not only identifies risks but also strengthens protective mechanisms across levels, from parent–child relationships to community supports and public policy. Doing so honors the complexity of children’s lived experiences while advancing the goal shared across all these studies: enabling every child to grow in environments that promote health, competence, and well-being.
